# Mitogen-Activated Protein Kinase Kinase 4 (MAP2K4) Promotes Human Prostate Cancer Metastasis

**DOI:** 10.1371/journal.pone.0102289

**Published:** 2014-07-14

**Authors:** Janet M. Pavese, Irene M. Ogden, Eric A. Voll, Xiaoke Huang, Li Xu, Borko Jovanovic, Raymond C. Bergan

**Affiliations:** 1 Department of Medicine, Northwestern University, Chicago, Illinois, United States of America; 2 Department of Preventative Medicine, Northwestern University, Chicago, Illinois, United States of America; 3 Robert H. Lurie Cancer Center, Northwestern University, Chicago, Illinois, United States of America; 4 Center for Molecular Innovation and Drug Discovery, Northwestern University, Chicago, Illinois, United States of America; China Medical University, Taiwan

## Abstract

Prostate cancer (PCa) is the second leading cause of cancer death in the US. Death from PCa primarily results from metastasis. Mitogen-activated protein kinase kinase 4 (MAP2K4) is overexpressed in invasive PCa lesions in humans, and can be inhibited by small molecule therapeutics that demonstrate favorable activity in phase II studies. However, MAP2K4's role in regulating metastatic behavior is controversial and unknown. To investigate, we engineered human PCa cell lines which overexpress either wild type or constitutive active MAP2K4. Orthotopic implantation into mice demonstrated MAP2K4 increases formation of distant metastasis. Constitutive active MAP2K4, though not wild type, increases tumor size and circulating tumor cells in the blood and bone marrow. Complementary *in vitro* studies establish stable MAP2K4 overexpression promotes cell invasion, but does not affect cell growth or migration. MAP2K4 overexpression increases the expression of heat shock protein 27 (HSP27) protein and protease production, with the largest effect upon matrix metalloproteinase 2 (MMP-2), both *in vitro* and in mouse tumor samples. Further, MAP2K4-mediated increases in cell invasion are dependent upon heat shock protein 27 (HSP27) and MMP-2, but not upon MAP2K4's immediate downstream targets, p38 MAPK or JNK. We demonstrate that MAP2K4 increases human PCa metastasis, and prolonged over expression induces long term changes in cell signaling pathways leading to independence from p38 MAPK and JNK. These findings provide a mechanistic explanation for human studies linking increases in HSP27 and MMP-2 to progression to metastatic disease. MAP2K4 is validated as an important therapeutic target for inhibiting human PCa metastasis.

## Introduction

Prostate cancer (PCa) is the most commonly diagnosed cancer in United States men, and the second most common form of cancer death [1]. While over 90% of individuals with localized PCa will not die from their disease, those with metastatic disease have a terminal diagnosis and the vast majority will die from PCa [2]. Understanding how the metastatic spread of human PCa is regulated is of critical biological importance. This knowledge will allow us to identify patients at risk, and thus in need of intervention, and will provide the basis for the development of targeted therapeutic strategies.

Mitogen-activated protein kinase kinase 4 (MAP2K4, also known as MKK4, MEK4, or SEK1) is a dual-specificity protein kinase that phosphorylates serine and threonine, as well as tyrosine residues. MAP2K4 is a member of the mitogen-activated protein kinase (MAPK) signaling pathway and typically activates two downstream targets, p38 mitogen-activated protein kinase (p38 MAPK) and c-Jun N-terminal kinase (JNK) [3]. The role of MAP2K4 in human PCa cancer progression, and the development of metastasis in particular, is controversial. MAP2K4 is located on chromosomal segment 17p11.2, which can be lost at a rate of approximately 7–10% in human epithelial cancers, particularly ovarian and breast cancers [4], [5] For this reason, it was initially presumed to be a tumor suppressor. In a rat PCa model, using cells lacking a chromosomal segment containing MAP2K4, specific restoration of MAP2K4 protein reduced PCa metastasis to the lung following flank injection of these cells [6]. In that model, increased MAP2K4 also delayed growth of metastatic cells arriving at the lungs, likely due to G1 cell cycle arrest [7].

However, other studies indicate that MAP2K4 imparts a pro-metastatic phenotype, and support the notion that it would increase metastasis. MAP2K4 activates p38 MAPK, which drives many steps of the metastatic cascade, including epithelial to mesenchymal transition (EMT), cellular invasion, and metastatic colonization (reviewed in [8]). MAP2K4 expression is increased in high grade prostatic intraepithelial neoplasia (HGPIN) lesions in both the murine-based TRAMP model of spontaneous PCa, as well as in human specimens [9]. Also, MAP2K4 expression is increased in early invasive, i.e., PCa, lesions in humans, and increased MAP2K4 expression significantly correlates with higher pathological stage [9]. Interestingly, in these studies and others, levels of MAP2K4 were then decreased in late stage metastasis, indicating that MAP2K4 increase is critical for early steps in the metastatic cascade [10]. This influence on early steps is confirmed in *in vitro* studies. Using several different human normal and cancer prostate cell lines and transient engineered expression of MAP2K4, our group demonstrated that MAP2K4 increases cell invasion, a critical indication of metastatic progression *in vitro* [11]. We also identified MAP2K4 as an important therapeutic target of small compound inhibitors. Specifically, we demonstrated that MAP2K4 was the therapeutic target of the small compound, 4′,5,7-trihydroxyisoflavone (genistein), for its effects upon invasion inhibition [11], [12], and that genistein inhibits human PCa metastasis in an orthotopic murine model [13]. Through a series of related studies, we identified the pathway, transforming growth factor (TGF)β → MAP2K4 → p38 MAPK → heat shock protein 27 (HSP27) → matrix metalloproteinase type 2 (MMP-2) → human PCa cell invasion, and that it is inhibited by genistein [11]–[16]. We also demonstrated that prospective genistein administration to humans with localized PCa decreases MMP-2 expression in prostate tissue [11]. Using *in vivo* animal models, altered MAP2K4 expression can alter metastasis in other human epithelial cancers. Particularly, other groups have shown MAP2K4 knockdown decreases metastatic tumor growth in mouse models of breast and pancreatic cancer [17], [18].

Given MAP2K4's altered expression and prognostic relevance in humans, its *in vitro* effects upon human prostate cells, and varied responses in rat and human epithelial cancer cell lines, it is important to specifically determine MAP2K4's role in regulating the metastatic behavior of human PCa. Although MAP2K4 is a therapeutic target of genistein, genistein exerts many different effects. Therefore despite genistein's inhibition of human PCa metastasis, the role of MAP2K4 in regulating metastasis formation cannot be determined from these findings. The uncertainty of MAP2K4's role in regulating human prostate cancer metastasis is further increased by studies across several different cancer types which support either a metastasis suppressor [6], [7], [19]–[21], or stimulatory role [9], [11], [17], [18]. Importantly, none have examined MAP2K4's role in regulating metastatic behavior of human PCa.

In the current study, we demonstrate several novel findings. First, MAP2K4 increased human PCa metastasis. MAP2K4 increased tumor growth *in vivo*, but not *in vitro*. Under the pressure of chronic expression, which emulates the clinical situation, MAP2K4 led to a rewiring of cell signaling pathways. This resulted in a bypass of immediate downstream signaling proteins, p38 MAPK and JNK. We identified a unique mechanism of compensation whereby MAP2K4 led to an up regulation in the expression of HSP27 total protein. This in turn resulted in higher absolute levels of phosphorylated HSP27, and increases in downstream MMP-2. The MAP2K4-driven metastatic phenotype was shown to be dependent upon HSP27 and its downstream effector, MMP-2. These findings demonstrate that MAP2K4 is an important driver of human PCa metastasis, and they thereby validate it as a therapeutic target for inhibiting PCa metastasis. They also provide a mechanistic explanation for clinical studies linking increases in MAP2K4, HSP27 and MMP-2 expression to the future development of metastasis.

## Materials and Methods

### Ethics Statement

All animal studies were carried out in strict accordance with the recommendations in the Guide for the Care and Use of Laboratory Animals of the National Institutes of Health. The protocol was approved by the Institutional Animal Care and Use Committee at Northwestern University (PHS Assurance #A3283-01). All surgery was performed under isoflurane anesthesia, and all efforts were made to minimize suffering.

### Cell Culture and Transfection

PC3-M and LNCaP cells were maintained in RPMI 1640 media supplemented with 2 mM L-glutamine, 10 mM HEPES buffer, 50units/ml penicillin, 50 µg/ml streptomycin, and 10% fetal bovine serum (Life Technologies) at 37°C in a humidified atmosphere of 5% carbon dioxide. 1542CPTX cells were maintained in Keratinocyte SFM media supplemented with 2 mM L-glutamine, 10 mM HEPES buffer, 50units/ml penicillin, 50 µg/ml streptomycin, 10% fetal bovine serum, 5 ng/ml EGF Human Recombinant, 50 µg/ml Bovine Pituitary Extract (Life Technologies) at 37°C in a humidified atmosphere of 5% carbon dioxide.

MAP2K4 stable transfected cells were created by transfecting PC3-M, LNCaP, or 1542CPTX cells, whose origin was previously described [22], with TransIT-LT1 Transfection Reagent (Mirus Bio LLC), and plasmid DNA per manufacturer's instructions. Cells were grown under G418 selection conditions, individual colonies harvested and expanded, as previously described by us [23]. In indicated experiments, resultant individual clonal cell lines were additionally transfected, as above, with GFP expression plasmid, selected under blasticidin growth conditions, and then purified using sterile cell sorting based upon GFP status. Cell lines were authenticated according to methods described in the American Type Culture Collection (ATCC) Technical Bulletin No. 8, Cell Line Verification Test Recommendations [24]. Specifically, cells from low passage (i.e., <15 passages) frozen stocks were used and were replenished after 20 passages, cells underwent routine microscopic examination to confirm uniform and standard cellular architecture and no microbial infection, and cells were tested and found negative for mycoplasma infection.

For knockdown experiments, Dharmacon ON-Targetplus SMARTpool siRNA targeting MAP2K4 (L-003574-00-0005), HSP27 (L-005269-00-0005), MMP-2 (L-005959-00-0005), or non-targeting siRNA (D-001810-10-05) was transfected into cells using Dharmafect Duo (T-2010, all from Thermo Scientific), along with pCMV-β-Galactosidase (Agilent Technologies), and used after 48 hours, as described by us [11].

### Reagents and Plasmids

The following were purchased: antibodies, SEK1/MKK4 (#9152), phospho-p38 MAPK (T180/Y182) (#4631), p38 MAPK (#9212), phospho-SAPK/JNK (T183/Y185) (#4668), SAPK/JNK (#9258), phospho-HSP27 (#2401), HSP27 (#2402), and GAPDH (#2118), all from Cell Signaling Technology, and horseradish peroxidase-conjugated anti-mouse and anti-rabbit, from GE Healthcare Biosciences; plasmids, wild type and constitutive active MAP2K4 (#14615 and #14813; Addgene) and GFP (Vivid Colors pcDNA 6.2/N-EmGFP-GW/TOPO, Invitrogen); chemical inhibitors, p38 MAPK inhibitor, SB203580 (Sigma-Aldrich) and associated negative control, SB202474, and JNK Inhibitor II (SP600125), and JNK Inhibitor II Negative Control (N^1^-Methyl-1,9-pyrazoloanthrone), all from EMD Millipore.

### Murine Model of Human Prostate Cancer Metastasis

Cells were implanted and distant metastasis quantified as previously described by us, with modifications [13], [25]. Briefly, 2.5×10^5^ cells were implanted into the ventral prostate of 6–8 week old male Balb/c athymic mice, fed Harlan Teklad 2016S chow and water *ad libitum*, and housed in a barrier facility with 12 hour light/dark cycles. After 6 weeks, tumor tissue was snap frozen, lungs were formalin fixed/paraffin embedded, and blood and bone marrow were cultured in complete RPMI 1640 media with G418 for 10 days, and presence of CTCs recorded.

With 45 micron step-sections, adjacent 4-micron lung tissue sections were immunostained for GFP and with hematoxylin and eosin (H&E). Metastases were scored, in a blinded fashion, as groups of ≥5 GFP positive cells.

### Cell Invasion Assays

Cell invasion was measured as described by us, with modifications [26]. After adding 1×10^5^ cells, in RPMI 1640 with 0.1% BSA, to BD BioCoat Growth Factor Reduced Matrigel chambers with 8 µm membranes, cells invaded for 24 hours toward the lower chamber, containing serum-free NIH 3T3 conditioned media. Assays were conducted at least 3 separate times, each in replicates of N = 3. In some experiments, cells were treated with 10 µM inhibitor 48 hours prior to, and during invasion.

Invading cells, i.e., on the bottom membrane, were methanol fixed, crystal violet stained, and imaged and counted under light microscopy. In parallel wells, cells on the top membrane were not removed, allowing confirmation of equal numbers of total cells loaded. For siRNA experiments, cells were stained using the *In Situ* β-Galactosidase Staining Kit (Agilent Technologies), followed by imaging and counting invaded and non-invaded β-gal positive cells per well.

### Cell Migration Assays

Cellular migration was measured as described by us, with modifications [26]. Each BD Falcon well, with 8 µm membranes, was loaded with 2.5×10^4^ cells, and allowed to migrate for 8 hours toward conditioned media in the lower chamber. Cells were stained with crystal violet, as above. Assays were conducted 3 separate times, each in replicates of N = 3.

### Quantitative Reverse Transcriptase Polymerase Chain Reaction

Quantitative reverse transcriptase polymerase chain reaction (qRT/PCR) was performed as described by us [27]. Briefly, RNA was isolated with RNeasy Mini Kit (Qiagen) for cells and with Trizol (Invitrogen) followed by RNeasy Mini Kit purification for tumor tissue, cDNA synthesized using TaqMan Reverse Transcription Reagents, and qPCR performed using TaqMan Universal PCR Master Mix and primer-probe pairs on a 7500 ABI Real Time PCR System (all from Life Technologies). Primer probes were: GAPDH (Hs99999905_m1), MMP-2 (HS00234422_m1), MMP-9 (Hs00234579_m1), MMP-10 (Hs00233987_m1), MMP-13 (Hs00233992_m1). At least two separate assays were performed, each in replicates of N = 2, resultant mean threshold cycles were normalized to GAPDH, and relative gene expression calculated by the 2^−ΔΔCt^ method [28].

### Cell growth assay

Five day cell growth assays were performed as described by us [23]. Briefly, after five days of non-confluent exponential growth in 96 well plates, dimethylthiazoldiphenytetrazolium bromide (MTT) was added, and OD_540_ determined. Assays were repeated three times, each in replicates of N = 3.

### Soft Agar Growth Assay

In a 6 well plate format, 1×10^4^ cells/0.5 ml 0.3% DNA grade base agar (Sigma Aldrich) were layered onto 1 ml of 0.5% agar, all made with and finally topped with 0.5 ml RPMI 1640 cell culture media. After 14 days, colonies were crystal violet stained and counted under light microscopy. Assays were conducted three times, each in replicates of N = 2.

### Western Blots

Western blots were performed as previously described by us [15]. Antibody concentrations were per manufacturer's instructions.

### Statistical analysis

Groups were considered statistically significant if the two-sided Student's t-test p-value was ≤0.05.

## Results

### MAP2K4 Increases Human Prostate Cancer Metastasis

**Figure 1:**
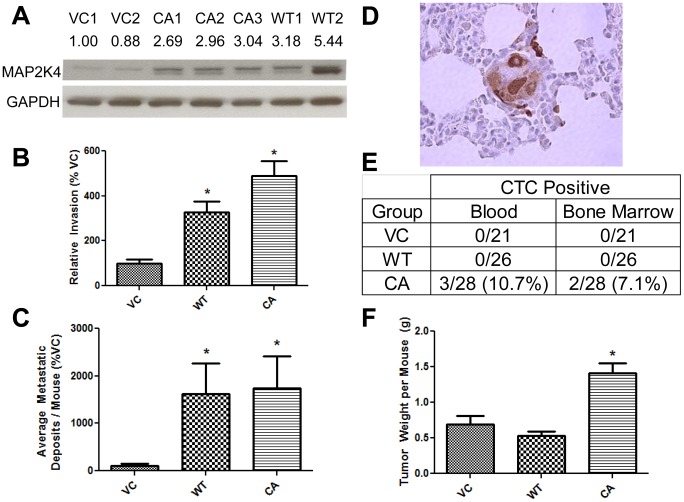
MAP2K4 Overexpression Promotes Human Prostate Cancer Cell Invasion and Metastasis. **A**) MAP2K4 protein expression in MAP2K4 variant cell lines. The expression of MAP2K4 protein was measured in wild type, WT1, WT2, and constitutive active, CA1, CA2, CA3, MAP2K4 over expressing cell lines, and vector control, VC1, VC2, cell lines by Western blot. A representative Western blot is depicted, with quantification of the MAP2K4/GAPDH levels, normalized to VC1 cells, shown above each lane. **B**) Invasion of MAP2K4 variant cell lines. Boyden chamber Matrigel cell invasion assays were performed, as described in Methods. Data are from 5 independent experiments, each in replicates of N = 3. Data from the respective wild type, constitutive active and vector control cell lines were combined to give an average read out for WT-MAP2K4, CA-MAP2K4 and VC cells, respectively. Data are expressed as the mean ± SEM percentage of VC cells. **C-E**) The effect of MAP2K4 expression upon metastatic spread and tumor growth. Equal numbers of mice were orthotopically implanted with VC1 or VC2 cells (forming the VC cohort), WT1 or WT2 cells (WT cohort), or with CA1, CA2 or CA3 cells (CA cohort). In (C) the resultant number of distant metastasis is expressed as the mean ± SEM percentage of VC mice, in (E) the number of mice developing circulating tumor cells is depicted, and in (F) tumor volume is graphed as the mean ± SEM percentage of VC mice. A representative image of a distinct metastasis is shown in (D). * denotes p≤0.05 between VC and an experimental group.

We engineered stable expression of wild type MAP2K4 (WT-MAP2K4), constitutive active MAP2K4 (CA-MAP2K4), or empty vector, for controls (VC), in PC3-M cells. The CA-MAP2K4 construct has Ser220Glu and Thr224Glu point mutations in its activation motif [29]. As determined by Western blot, Fig 1A, CA-MAP2K4 and WT-MAP2K4 cell lines express MAP2K4 2.9 and 4.3-fold higher levels on average, respectively, compared to the average for VC cell lines. Each individual CA- or WT-MAP2K4 cell line expresses levels ≥2.6-fold higher. These findings demonstrate that it is possible to engineer MAP2K4 overexpressing cell lines.

To evaluate the functional relevance of MAP2K4 overexpression, we assessed the ability of cell lines to invade in a Matrigel Boyden chamber assay, Fig 1B. Overexpression of MAP2K4 significantly increased invasion to a mean of 328% for WT-MAP2K4 cell lines and to 490% for CA-MAP2K4 cell lines, compared to VC cell lines. For individual cell lines, i.e., two of two WT-MAP2K4 and three of three CA-MAP2K4, invasion levels were statistically significant, compared to control cells (data not shown). In parallel with engineering MAP2K4 overexpressing cell lines, we also engineered a series of stable MAP2K4 knockdown cell lines using two different short hairpin shRNA expressing vectors, and confirmed knockdown was specific to MAP2K4 (data not shown). However, when testing the function of these cell lines in invasion assays, we did not observe a consistent phenotype. Specifically, half of the clonal cell lines exhibited decreased invasion, while half showed no change. This finding is congruent with a threshold level of expression required for effects by MAP2K4 upon regulation of invasion. Due to lack of consistent phenotype with knockdown of MAP2K4, we elected not to pursue this further. In contrast, with MAP2K4 overexpression our findings clearly demonstrate a consistent phenotype of increased invasion and are consistent with observations in humans, where increased MAP2K4 expression occurs in invasive lesions in the prostate.

To determine whether increased MAP2K4 expression increases metastatic potential, we examined the effect of MAP2K4 upon tumor growth and metastasis formation *in vivo* using an orthotopic murine model of human PCa metastasis specifically developed by us to quantify these parameters [13], [25]. This model is responsive to anti-motility therapeutic agents as well as to alterations in expression of genes that regulate metastatic progression. In this model, human PCa cells are directly implanted into the prostate, allowing for measurement of primary tumor size, PCa cells entering the blood stream as in-transit circulating tumor cells (CTCs), PCa cells in the bone marrow, and of distant lung metastasis. Metastasis to the lungs is a primary endpoint feature of this model, allowing accurate quantification of individual metastatic deposits, and therefore an accurate measure of the ability of primary cells to metastasize out of their organ of origin, emulating the situation in humans.

Three cohorts of mice were implanted with either VC cells (where equal numbers of mice were implanted with either VC1 or VC2 cells), WT-MAP2K4 cells (equal numbers implanted with WT1 or WT2 cells), or with CA-MAP2K4 cells (equal numbers implanted with either CA1, CA2 or CA3 cells), yielding 21, 26 and 28 mice viable mice after surgery in VC, WT-MAP2K4 and CA-MAP2K4 cohorts, respectively. Six weeks after surgery, primary tumor size, metastasis, and CTCs were measured in each mouse. Increased expression of either WT-MAP2K4 or CA-MAP2K4 significantly increased distant metastasis by 16.2 and 17.4-fold, respectively, compared to VC mice, with no significant difference between WT-MAP2K4 and CA-MAP2K4 mice, Fig 1C. A representative image of a distinct metastasis is shown in Fig 1D. These findings demonstrate increased MAP2K4 increases human PCa metastasis. Interestingly, CTCs were only observed in CA-MAP2K4 mice, were detected in the blood of 11% (3/28) and bone marrow of 7% (2/28), Fig 1E, and demonstrate that CTCs are relatively uncommon in MAP2K4-driven metastasis.

Despite WT-MAP2K4 mice exhibiting significantly increased metastasis compared to VC, the size of primary tumor in WT-MAP2K4 mice was an average of 24% smaller, though not statistically significant, Fig 1F. As increased tumor size in-and-of-itself can affect metastatic spread, this demonstrates MAP2K4 has a primary effect upon the regulation of metastatic behavior. Interestingly, tumors of CA-MAP2K4 mice significantly increased by 2.04 and 2.67-fold, compared to VC and WT-MAP2K4 mice, respectively. Therefore, wild type MAP2K4 does not increase tumor growth, but constitutive active MAP2K4 does.

### MAP2K4 alters protease production, but not cell migration or cell growth *in vitro*

**Figure 2:**
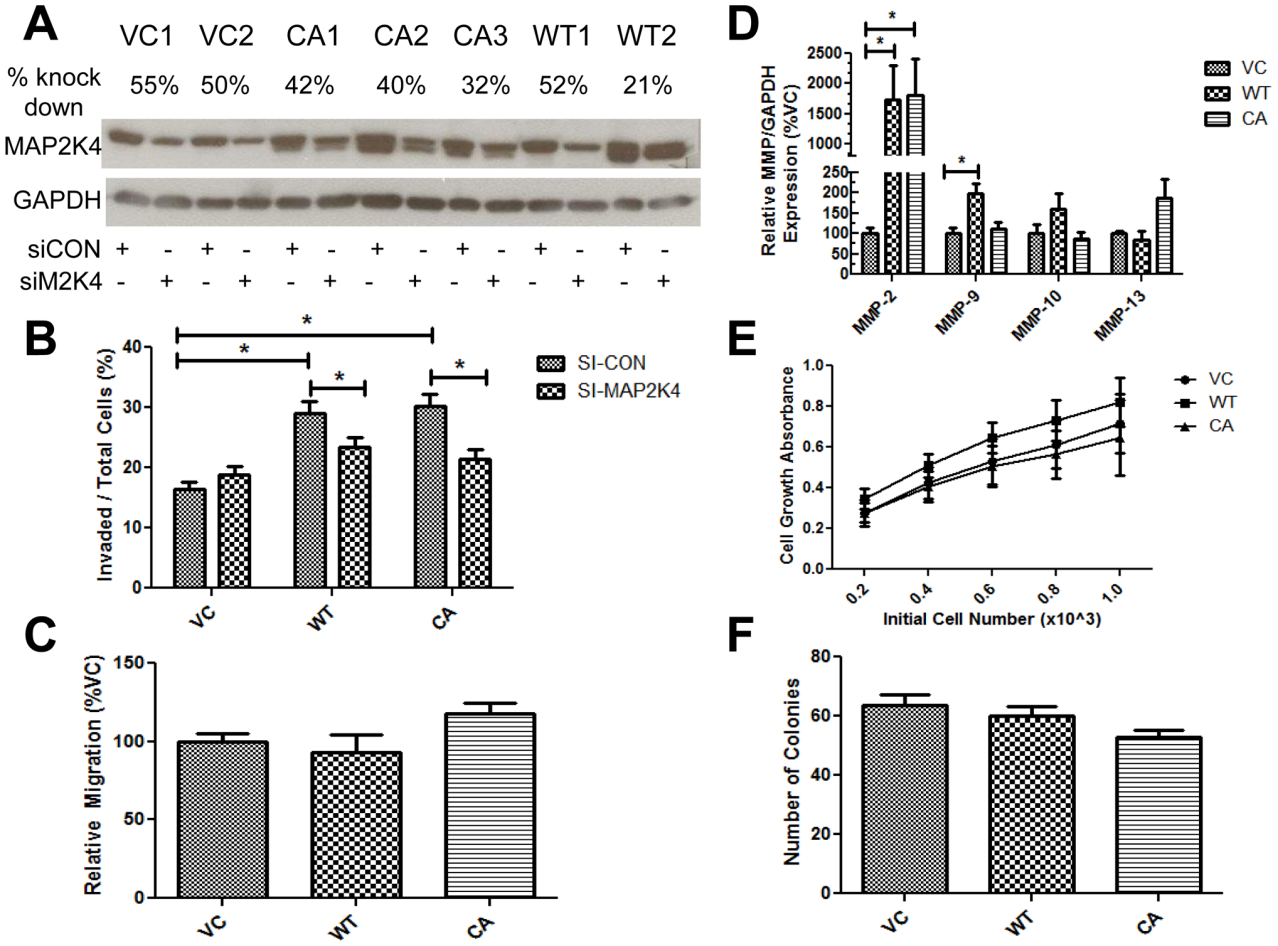
MAP2K4 alters protease production, but not cell migration or cell growth *in vitro*. Studies were conducted on MAP2K4 variant cell lines. **A**) Knockdown of MAP2K4 protein by siRNA. After transfection of cells with siRNA targeting MAP2K4 (siMAP2K4) or non-targeting control (siCON), MAP2K4 expression was measured by Western blot. Percent knockdown is denoted above the blot. **B**) Effect of MAP2K4 knockdown on cell invasion. MAP2K4 variant cell lines were treated with siRNA, as indicated, and cell invasion measured and depicted as in Fig 1. Data are from four experiments, each in replicates of N = 2, and are expressed as the percentage of invading cells. **C**) Effects on cell migration. Cell migration was measured as described in Methods. Data from individual types of clones were combined, are from three independent experiments, each in replicates of N = 3, and are expressed as the percentage of migrating cells, normalized to VC. **D**) MMP transcript expression. The levels of MMP-2, MMP-9, MMP-10 and MMP-13 transcript levels were measured by qRT/PCR, normalized to GAPDH, and expressed as the percentage of VC. Data are from four independent experiments, each in replicates of N = 2. **E**) Cell growth. Cells were plated at the indicated concentrations, allowed to grow for 5 days, MTT added, and optical density determined. Data are from three independent experiments, each N = 3. **F**) Colony formation. Colony formation after 14 days was determined in three independent experiments, each N = 2, and expressed as the percent of VC. Values in all graphs are the mean ± SEM. * denotes p≤0.05 between the indicated groups.

Having shown that MAP2K4 increases metastasis, we sought to deepen our understanding of the underlying mechanisms. Chronic MAP2K4 expression increased cell invasion (Fig 1B), and cell invasion is a major determinant of metastatic behavior. We next evaluated whether increased cellular invasion in MAP2K4 overexpressing cells was dependent on MAP2K4 by knocking down MAP2K4 with siRNA. In Fig 2A, for each of the cell lines, knockdown of 21–55% was achieved. We next demonstrated that MAP2K4 knockdown significantly reversed the increased invasion phenotype observed in both WT-MAP2K4 and CA-MAP2K4 cells, Fig 2B. Therefore, cell invasion remains dependent upon continued MAP2K4 expression.

Given the recognized role of cell invasion in metastatic progression, we further examined the mechanistic underpinnings of MAP2K4's effect upon cell invasion. Cell invasion is primarily driven by cell migration coupled to protease digestion. Neither WT-MAP2K4 nor CA-MAP2K4 overexpression increased cell migration in an uncoated Boyden chamber assay, Fig 2C. Additionally, we observed no consistent effect on cell migration measured by single-cell motility assay (data not shown). We next examined MAP2K4's effect upon protease expression. Because there is a wide array of known proteases, we first conducted a screen for relevant proteases. We previously established genistein binds to and inhibits MAP2K4 kinase, thereby inhibiting human PCa cell invasion [11]. We therefore examined the effects of genistein treatment on native PC3-M cells using the Extracellular Matrix and Adhesion Molecules RT^2^ Profiler PCR Array system, which profiles 84 human genes regulating cell-cell and cell-matrix interactions (Figure S1). The following proteases were significantly down-regulated by at least 2-fold by genistein: MMP-2, MMP-10, and MMP-13, and were confirmed by gene-specific qRT/PCR (Figure S1). Based upon these findings, we examined expression of these three proteases in the context of MAP2K4 overexpression. Additionally, as MMP-2 and MMP-9 are closely related gelatinases, frequently co-regulated, and as genistein affects MMP-9 is some cell lines, albeit episodically and to a small degree, we additionally measured MMP-9 [30]. Measurement of transcript levels by qRT/PCR revealed that MMP-2 was significantly increased by a mean of >15-fold compared to VC, in both WT-MAP2K4 and CA-MAP2K4 cells. MMP-9 did significantly increase 2-fold, but only in WT-MAP2K4 cells. MMP-10 and MMP-13 were unaffected. Therefore, MAP2K4-mediated increases in invasion are not due to changes in cell migration, but are associated with changes in protease expression, MMP-2 in particular. This is clinically relevant as MMP-2 can degrade collagen IV, a major component of prostate tissue, and has been associated with poor prognosis, including the development of metastasis (reviewed in [31]).

We next investigated the increased tumor size observed in CA-MAP2K4 mice, Fig 1E, to determine if growth is altered *in vitro*, Fig 2E-F. In a five day *in vitro* MTT assay, no differences in cell growth between groups were observed, Fig 2E. In a 14-day soft agar colony formation assay, CA-MAP2K4 cells grew slightly slower than VC and WT-MAP2K4 cells, but this change was not significant, Fig 2F. These findings demonstrate that under *in vitro* growth conditions, neither WT-MAP2K4 nor CA-MAP2K4 affect cell growth.

### MAP2K4 overexpression specifically alters HSP27 phosphorylation and total protein expression

The above findings demonstrate that MAP2K4 relatively specifically increases MMP-2. Transient modification of MAP2K4 regulates MMP-2 and cell invasion by phosphorylation and activation of p38 MAPK [12]. In addition to p38 MAPK, MAP2K4 can also either activate JNK, or both proteins, dependent upon the model system [32]. JNK is commonly associated with changes in proliferation and apoptosis in response to stress in human PCa [33]–[35], but also with changes in cellular migration and invasion [36], [37]. We therefore hypothesized that chronic MAP2K4 expression would increase activation of p38 MAPK and/or JNK. We tested this theory by examining phospho-p38 MAPK, -JNK1 and -JNK2 levels by Western blot in MAP2K4 over expressing cell lines, using phosphoprotein specific antibodies. However, our findings did not support our hypothesis as overexpression of MAP2K4 did not increase either phospho- or total protein levels of either p38 MAPK or JNK, Figure S2.

**Figure 3:**
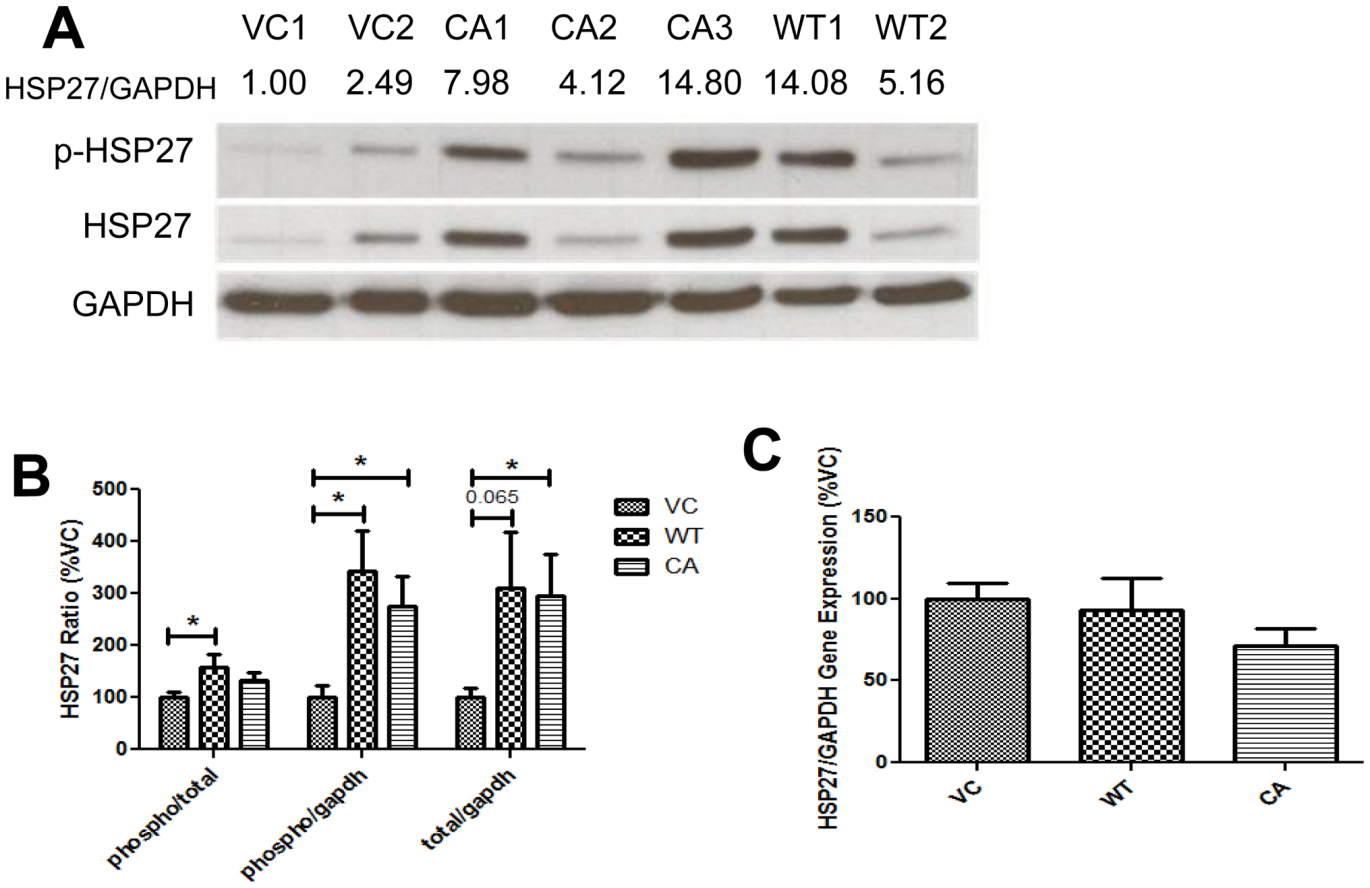
MAP2K4 overexpression specifically alters HSP27 phosphorylation and total protein expression. The expression of total and phosphorylated forms of HSP27 proteins were assessed by Western blot in the indicated cell lines, **A-B**. Data from three separate blots are graphically depicted in**B**, as mean ± SEM. **C**), HSP27 transcript levels were measured by qRT/PCR, normalized to GAPDH, and expressed as the mean ± SEM percentage of VC. Data are from three independent experiments, each in replicates of N = 2. * denotes p≤0.05 between the indicated groups.

Our findings supported the hypothesis that under conditions of sustained MAP2K4 overexpression, as occurs in the clinical scenario, compensatory pathways may be bypassing targets immediately downstream of MAP2K4, thereby leaving them in an inactivated state. HSP27, a downstream of p38 MAPK, increases both MMP-2 expression and cell invasion in human PCa, under conditions of transient transfection [12], [15]. We therefore examined the status of HSP27, Fig 3A-B. Overall levels of both phospho- and total HSP27, as compared to GAPDH, were increased. Phospho- and total HSP27 increased by 3.4 (significantly) and 2.7 (only a trend; 2-sided p-value  =  0.065), respectively, for WT-MAP2K4 cells, and both significantly by 3.1 and 3.0-fold for CA-MAP2K4 cells. The phospho-HSP27/total HSP27 ratio increase was small, and significant only for WT-MAP2K4 cells. These findings demonstrate MAP2K4 leads to increased levels of total and phospho-HSP27 protein. There were no differences in HSP27 gene expression between MAP2K4 overexpressing and VC cells, Fig 3C, indicating that differences in protein expression stemmed from alterations in translation and/or protein degradation. Taken together, these findings determine that chronic MAP2K4 overexpression leads to increased HSP27 protein expression, while bypassing the activation of p38 MAPK and JNK.

### MAP2K4 increases cellular invasion, MMP-2 production, and HSP27 in early stage PCa cell lines

**Figure 4:**
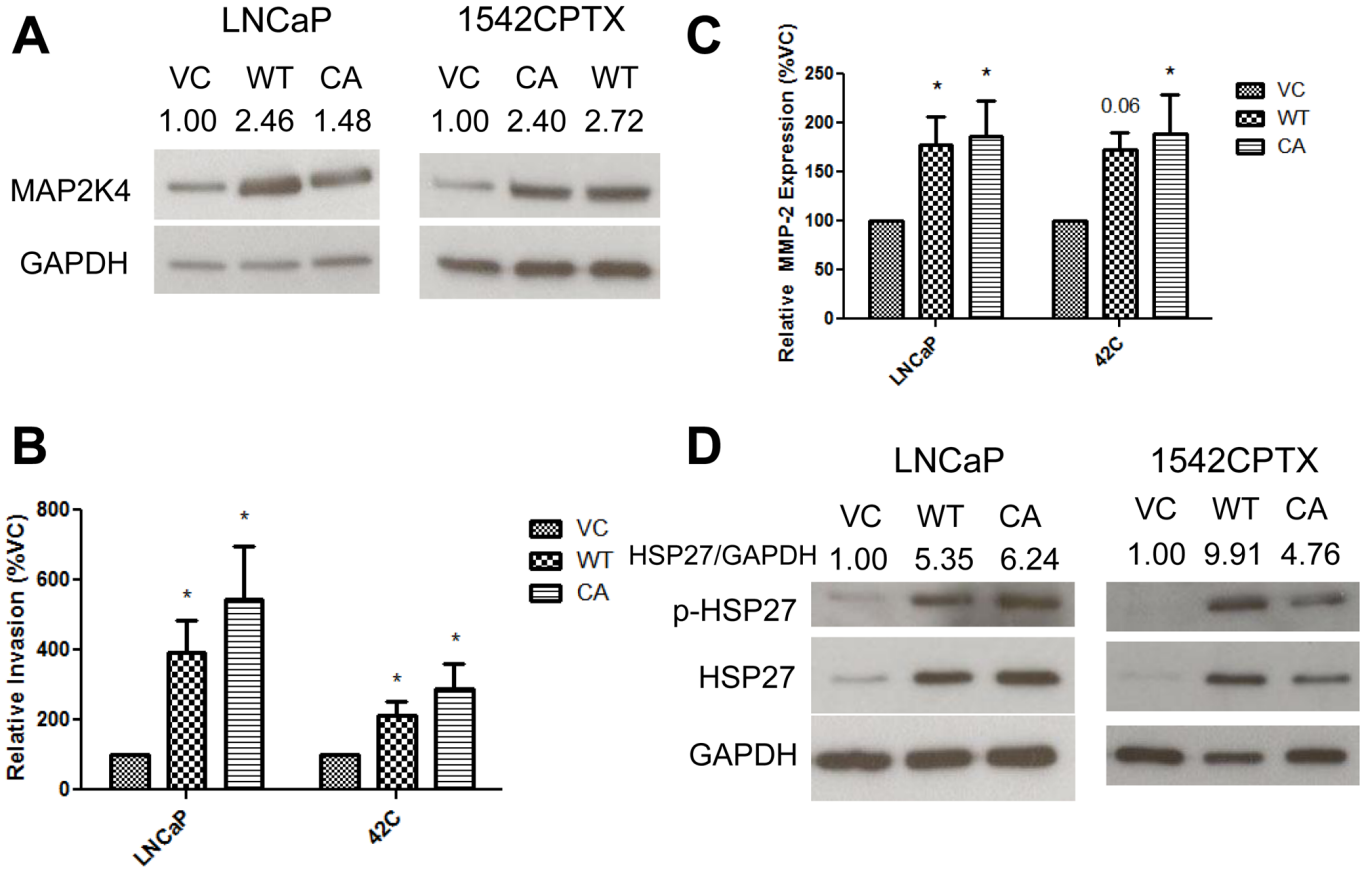
MAP2K4 increases cellular invasion, MMP-2, and HSP27 in early-stage cancer cell lines. **A**) MAP2K4 protein expression in LNCaP and 1542CPTX cell lines by Western Blot. **B**) Relative cellular invasion of LNCaP and 1542CPTX (42C) cell lines as measured by a Matrigel coated Boyden chamber. Data are from four independent experiments, each in replicates of N = 2. * denotes p≤0.05 between the indicated groups. **C**) Relative MMP-2 mRNA expression in LNCaP and 42C cell lines as measured by qRT/PCR. Data are from five independent experiments, each in replicates of N = 2. * denotes p≤0.05 between the indicated groups. **D**) Phosphorylated and total HSP27 expression in LNCaP and 1542CPTX cell lines by Western Blot.

In addition to the PC3-M cell line, we also created stable cell lines using the LNCaP and 1542CPTX (42C) cancer cell lines. Compared to PC3-M, LNCaP and 42C represent earlier stage PCa. For these experiments we created a clonal pool of cells that stably overexpress MAP2K4. Their overall MAP2K4 expression was between 1.5 and 2.7 fold increased as measured by Western Blot, Fig 4A. We next measured the relative cellular invasion levels of these cell lines. Congruent with the findings in Fig 1B, both the LNCaP and 42C MAP2K4 overexpressing cells significantly increase cellular invasion, Fig 4B. In MAP2K4 overexpressing LNCaP cells, there was a 3.9–5.4 fold increase in cellular invasion, and in MAP2K4 overexpressing 42C cells, there was a 2.1–2.9 fold increase.

Having identified that MMP-2 is increased in PC3-M MAP2K4 overexpressing cell lines, we also measured the relative levels of MMP-2 in LNCaP and 42C cell lines. In both cell lines, there was a 1.8–1.9 fold increase in relative MMP-2 mRNA levels in these cell lines, Fig 4C. The increased MMP-2 expression was statistically significant in all inscantes, except in the 42C WT-MAP2K4 cells, where the increase represented a strong statistical trend (p-value  = 0.057). Finally, we also show an increase in both the phosphorylated and total levels of HSP27 protein in these cell lines, Fig 4D. LNCaP cells exhibited a 5.4–6.2 fold increase in HSP27 protein levels, and 42C cells a 4.8–9.9 fold increase. These findings, along with similar ones above in PC3-M cells, demonstrate under conditions of chronic MAP2K4 overexpression, cellular invasion, MMP-2 expression, and HSP27 all increase.

### MAP2K4 increases HSP27 and MMP-2 expression *in vivo*

**Figure 5:**
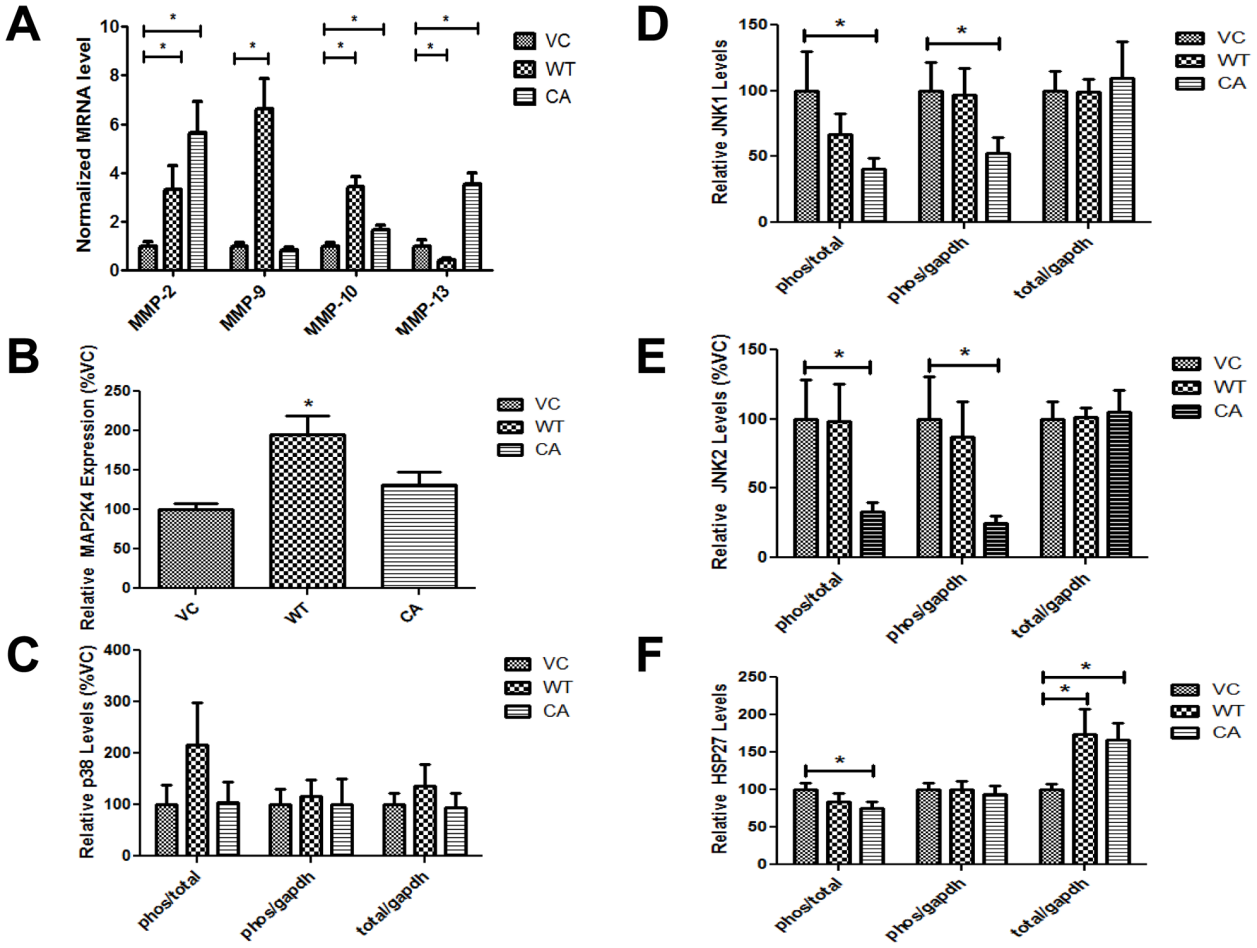
MAP2K4 increases HSP27 and MMP-2 expression *in vivo*. **A**) MMP transcript expression of tumor samples. The levels of MMP-2, MMP-9, MMP-10 and MMP-13 transcript levels were measured by qRT/PCR, normalized to GAPDH, and expressed as the mean ± SEM percentage of VC. Each tumor sample was run twice, in duplicates of N = 2, and compared to a reference sample. **B-F**) Quantification of phospho- and total protein expression in tumor samples. Protein expression was measured by Western Blot, twice for each tumor, compared to a reference sample, and graphed data are the mean ± SEM percent of VC. * denotes p≤0.05 between the indicated groups.

The relevance of increased total HSP27 is supported by increased HSP27 expression's association with poor patient outcome and development of metastasis in human PCa patients [38]. We therefore examined levels of MAP2K4 downstream protein activation and protease gene expression *in vivo* in tumor samples from mice, Fig 1. The pattern of protease gene expression, by qRT/PCR, was similar to that observed *in vivo* (compare Figs 5A and 2D): MMP-2 was significantly and strongly increased in WT-MAP2K4 and CA-MAP2K4 mice by 3.3 and 5.6-fold respectively, compared to VC mice, MMP-9 significantly increased only in WT-MAP2K4 mice, MMP-10 significantly increased by 3.4 and 1.7-fold, and MMP-13 significantly decreased by 2.4-fold and increased by 3.6-fold, Fig 5A. These findings demonstrate large and consistent effects upon MMP-2 by MAP2K4 across *in vitro* and *in vivo* systems.

Evaluation of protein expression in tumors, by Western blot, show increased MAP2K4 levels of 1.95 and 1.31-fold in WT-MAP2K4 and CA-MAP2K4 mice, respectively, compared to VC mice, but was only significant for WT-MAP2K4 mice. This higher expression pattern in WT-MAP2K4 compared to CA-MAP2K4 emulated that observed for cells at implantation (see Fig 1A). However, once implanted cells were not exposed to G418. There were no differences in either total or phospho-p38 MAPK protein between cohorts, Fig 5C. In contrast, levels of both phospho-JNK1 and phospho-JNK2 were significantly decreased in CA-MAP2K4 mice, but not in WT-MAP2K4 mice, compared to VC mice, Figs 5D-E. There were no changes in total JNK protein levels, and thus there was a significant decrease in phospho-JNK/total-JNK ratio, for both JNK1 and JNK2. Importantly, HSP27 protein was significantly increased by >1.7-fold in both WT-MAP2K4 and CA-MAP2K4 mice, Fig 5F, similar to *in vitro* findings. However in contrast to *in vitro* findings, phospho-HSP27 levels were not increased in tumors of MAP2K4 overexpressing mice. Phospho/total HSP27 levels were slightly decreased in WT-MAP2K4 mice, and significantly decreased in CA-MAP2K4 mice. Note that the interpretation of phospho-protein results is less informative than that of total protein [14], as the prostate expresses very high levels of prostatic acid phosphatase, which affects phosphoprotein levels post-tissue and cell harvest, and can do so in a protein-specific fashion. Together, findings in tumor tissue emulate those *in vitro* for total protein expression, with no changes p38 MAPK, JNK1 and JNK2 levels, but increased HSP27 expression in both WT- and CA-MAP2K4 mice. However, the phosphoprotein expression pattern in tumor differed from that *in vitro*, with a decrease in phospho-JNK1 and JNK2, and no increase in phospho-HSP27.

### MAP2K4's pro-invasive effects are dependent on HSP27 and MMP-2

**Figure 6:**
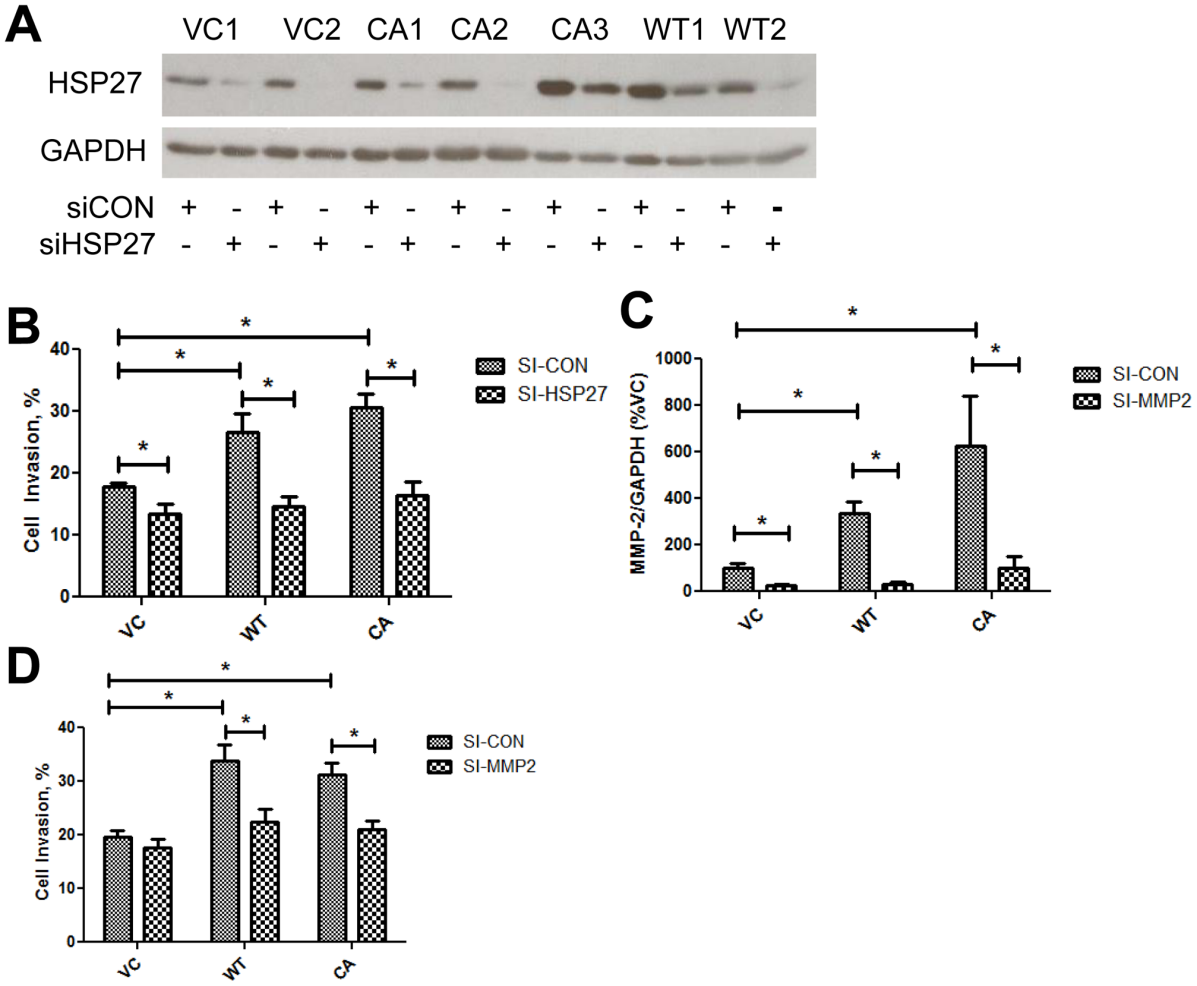
MAP2K4's pro-invasive effects are dependent on HSP27 and MMP-2. **A**) Knockdown of HSP27 protein by siRNA. After transfection of cells with siRNA targeting HSP27 (siHSP27) or non-targeting control (siCON), HSP27 expression was measured by Western blot. **B**) Effect of HSP27 knockdown on cell invasion. MAP2K4 variant cell lines were treated with siRNA, as indicated, and cell invasion measured and depicted as in Fig 1. Data are from four experiments, each in replicates of N = 2, and are expressed as the percentage of invading cells. **C**) Knockdown of MMP-2 mRNA by siRNA. After transfection of cells with siRNA targeting MMP-2 (siMMP2) or non-targeting control (siCON), MMP-2 expression was measured by qRT/PCR. **D**) Effect of MMP-2 knockdown on cell invasion. MAP2K4 variant cell lines were treated with siRNA, as indicated, and cell invasion measured and depicted as in Fig 1. Data are from four experiments, each in replicates of N = 2, and are expressed as the percentage of invading cells.

Findings above demonstrate increased HSP27 and MMP-2 are associated with increased MAP2K4 expression. We therefore investigated whether HSP27 and MMP-2 are necessary for MAP2K4-mediated invasion using the PC3-M derived cell lines. The ability of siRNA targeting HSP27 to knockdown HSP27 protein levels by ≥60% in all cell lines tested is shown in Fig 6A. HSP27 knockdown completely abrogated MAP2K4-induced increases in cell invasion in WT-MAP2K4 and CA-MAP2K4 cells, Fig 6B. Knockdown of MMP-2 transcript by ≥85% and ≥75% in MAP2K4 overexpressing and VC cells, respectively, is achieved, Fig 5C. MMP-2 knockdown completely abrogates MAP2K4-induced increases in invasion, in both WT-MAP2K4 and CA-MAP2K4 cells, Fig 6D.

In contrast, inhibition of immediate downstream targets p38 MAPK or JNK kinase activity, using the respective kinase-specific chemical inhibitors SB203580 and SP600125, had no significant impact upon MAP2K4-mediated increases in invasion, compared to associated inactive controls, SB202474 and JNK Inhibitor II Negative Control, respectively, Figure S2. Together, these findings demonstrate that MAP2K4-mediated increases in invasion are dependent upon HSP27 and MMP-2.

## Discussion

We have demonstrated for the first time that MAP2K4 increases human PCa metastasis. This validates the importance of MAP2K4 in human PCa metastatic progression. Findings from this study provide a mechanistic explanation for the clinical observation of increased MAP2K4 in invasive lesions in human PCa tissue [9]. In this regard, the model of metastasis used in the current study provides a measure of the cells' ability to escape the prostate gland, travel through the bloodstream, and implant and divide in a distant location, as in the clinical scenario. Importantly, this study also validates MAP2K4 as a biologically important target whose function can be inhibited by small molecule therapeutics. Given that small molecule therapeutics targeting MAP2K4 have been shown to inhibit cell invasion and metastasis of human PCa cells in preclinical studies, and that when tested in prospective human trials, those same therapeutics decrease MMP-2 expression in human prostate tissue, our current validation of MAP2K4 function *in vivo* provides essential information [11], [13]. Together, these findings provide the rationale to support dedicated future efforts designed to discover more effective novel therapeutics that target MAP2K4.

The clinical importance of the current findings is supported by demonstrating for the first time that overexpression of MAP2K4 increases HSP27 protein expression. Evaluation of human clinical samples has shown that HSP27 expression increases with PCa progression, and this increase is associated with development of metastatic disease [39]–[41]. Interestingly, we demonstrated MAP2K4 did not affect HSP27 transcript levels, consistent with regulation at the post-translational level. As HSP27 is a small heat shock protein, protein stabilization and degradation are both common regulators of the HSP family of chaperone proteins. Given HSP27's importance in regulating MAP2K4-mediated regulation of transformation to a metastatic phenotype, future studies are needed to investigate the mechanism by which MAP2K4 regulates HSP27 protein levels.

We also established for the first time that MMP-2 is necessary for MAP2K4-driven cell invasion and that MMP-2 is increased under chronic overexpression of MAP2K4 in cell lines and mouse tumors. In light of the large number of known proteases, and that many are affected by a given manipulation, including in the present system, our findings of specific dependence upon MMP-2 provides important mechanistic insight. Here too, the current findings directly link to the clinic. MMP-2 has long been implicated in metastatic transformation in a variety of cancer types including PCa. Increased expression of MMP-2 in primary PCa lesions in humans portends the future development of metastasis and death from PCa [42]–[45]. Finally, the importance of MAP2K4's effect upon MMP-2, a protease, in regulating cell invasion was further corroborated by the fact that MAP2K4 did not alter cell migration, which does not require protease action.

Of high importance, this study identified differences in signaling pathway function under conditions of chronic MAP2K4 expression, compared to those existing after transient alterations of MAP2K4 expression, as had been described by our group and others. Specifically, both p38 MAPK and JNK are immediate downstream effectors of MAP2K4 in multiple systems and have shown to be activated by increased MAP2K4 expression and activation by TGFβ. p38 MAPK and JNK can both modulate cell invasion, including our own work determining p38 MAPK regulates cell invasion in PC3-M cells [12], [36], [37], [46], [47]. In the current study we demonstrate that under conditions of chronic MAP2K4 overexpression, both JNK and p38 MAPK are bypassed, showing no activation under chronic MAP2K4 expression. In fact, in tumor samples, there is actually a decrease in JNK phosphorylation in CA-MAP2K4 cell lines, which is likely a compensatory response from other related pathways. It is likely that transient expression studies provide a measure of existing pathway function, while chronic expression provides a measure of cellular response to abnormal pathway activation over time. The latter situation emulates the clinical scenario, under which extended changes in protein expression occur. Further highlighting the importance of examining chronic changes in gene product expression, is our current finding that knockdown of MAP2K4 did not lead to a specific functional phenotype. This finding indicates that there is a threshold level of expression required for effects by MAP2K4 upon regulation of invasion under conditions of chronic alterations of expression. This differs from transient MAP2K4 knockdown, wherein we had demonstrated that decreased invasion is observed [11]. Together, these findings highlight the importance of evaluating long term changes in gene product expression when the ultimate goal is to understand biology in humans. Finally, these findings strengthen the role of both HSP27 and MMP-2 in the effects of MAP2K4 on cellular invasion, as the stable expression of these proteins drive these effects, corroborating observations in clinical samples.

Our model of human PCa metastasis allowed examination of circulating tumor cells (CTCs) in the blood and bone marrow. Given the overall low percentage of CTCs detected in the current study, definitive conclusions are inherently limited. This important consideration notwithstanding, it was interesting that CTCs were only detected in CA-MAP2K4 mice and were not frequent, similar to the clinical situation [48]. While circulation through the lymphatic system could provide an alternative route of dissemination, the predominance of lung metastasis in this model implicates hematologic-based dissemination. Currently the kinetics of CTC cell release is still largely not understood. It is possible that CTCs escape from tumors into the bloodstream through infrequent episodic bursts, and thus in the current model, blood was collected most frequently in the larger window of time in between these bursts. Interestingly, using the same parental cell line as in the current study, but engineered with a different gene, CTCs were observed at much higher levels, even in controls [25]. These findings coupled with the current ones suggest several factors likely contribute to differences in CTC frequency, and that some appear to be system dependent.

Despite WT-MAP2K4 and CA-MAP2K4 having similar effects upon metastasis, CA-MAP2K4 specifically and dramatically increased tumor size. Though a constitutive active construct ensured activation within the cell independent of additional stimuli, this is an artificial construct and MAP2K4 mutations in clinical samples are rare. Therefore, the relevance of these findings to the clinical situation is difficult to extrapolate. These important caveats notwithstanding, a decrease in both JNK1 and JNK2 phosphorylation was specific to CA-MAP2K4 cells. This finding clearly demonstrates WT-MAP2K4 and CA-MAP2K4 can differentially affect cell signaling. Further, it provides anecdotal findings that this may be mechanistically related. However, given the tenuous link of CA-MAP2K4 to the clinical scenario and its scope beyond the focus of the current study, we did not pursue these investigations at this time.

Taken together, we show for the first time that MAP2K4 expression increases human PCa metastasis. As MAP2K4's kinase activity can be inhibited by small molecule therapeutics, and this leads to inhibition of human PCa cell invasion and metastasis, our findings have validated the importance of MAP2K4 as a therapeutic target for inhibition of metastasis. Further, we demonstrate for the first time that MAP2K4-mediated transformation to an invasive, i.e., metastatic phenotype, is dependent upon HSP27 and MMP-2. Finally, we demonstrate signaling pathway activation and dependence differ in the context of chronic changes in MAP2K4 expression, compared to those observed after transient alterations. These findings suggest such effects need to be taken into consideration, and approaches to prognostication and design of therapeutic strategies may have inherent limitations if only based upon pathways defined in otherwise transiently-perturbed model systems.

## Supporting Information

Figure S1 Extracellular Matrix and Adhesion Molecules RT^2^ Profiler Array. **A**) PC3-M cells were treated with 10 µM genistein or DMSO control for 3 days, and the Extracellular Matrix and Adhesion Molecules RT^2^ Profiler PCR Array performed as per manufacturer's instructions three separate times. A ≥2 fold change with a two-sided Student's t-test p value ≤0.05 was considered significant. Seven genes of interest were identified: HGF, ITGA7, MMP-2, MMP-10, MMP-13, TIMP3, TSHR. **B**) Confirmation of MMP-2, MMP-10, and MMP-13 regulation by genistein by qRT/PCR. Cells were treated with 10 µM genistein or DMSO control for three days, and qRT/PCR performed using gene-specific primer/probe sets. Data are from three experiments, each in replicates of N = 2. * denotes p≤0.05 between control and experimental group.
(TIF)

Figure S2 Protein expression of p38 MAPK and JNK, Cellular invasion with p38 MAPK and JNK inhibitors. **A-C**) The phosphorylated and total protein levels of p38 MAPK, JNK1, and JNK2 as measured by western blot. These were performed in triplicate, quantified, and represented as mean ± SE. **D-E**) Relative cellular invasion following 48 treatment with chemical inhibitors SB203580 or SP600125, which inhibit p38 MAPK and JNK respectively, or their respective negative controls, SP202474 or JNK Negative Control II (N^1^-Methyl-1,9-pyrazoloanthrone). Data are from three experiments, each in replicates of N = 3. * denotes p≤0.05 between the indicated groups.
(TIF)
