# Supplementary figures and images for: Mitogen-Activated Protein Kinase Kinase 4 (MAP2K4) Promotes Human Prostate Cancer Metastasis
Source: PLoS One. 2014 Jul 14;9(7):e102289. doi: 10.1371/journal.pone.0102289 (PMC4096757; doi:10.1371/journal.pone.0102289)

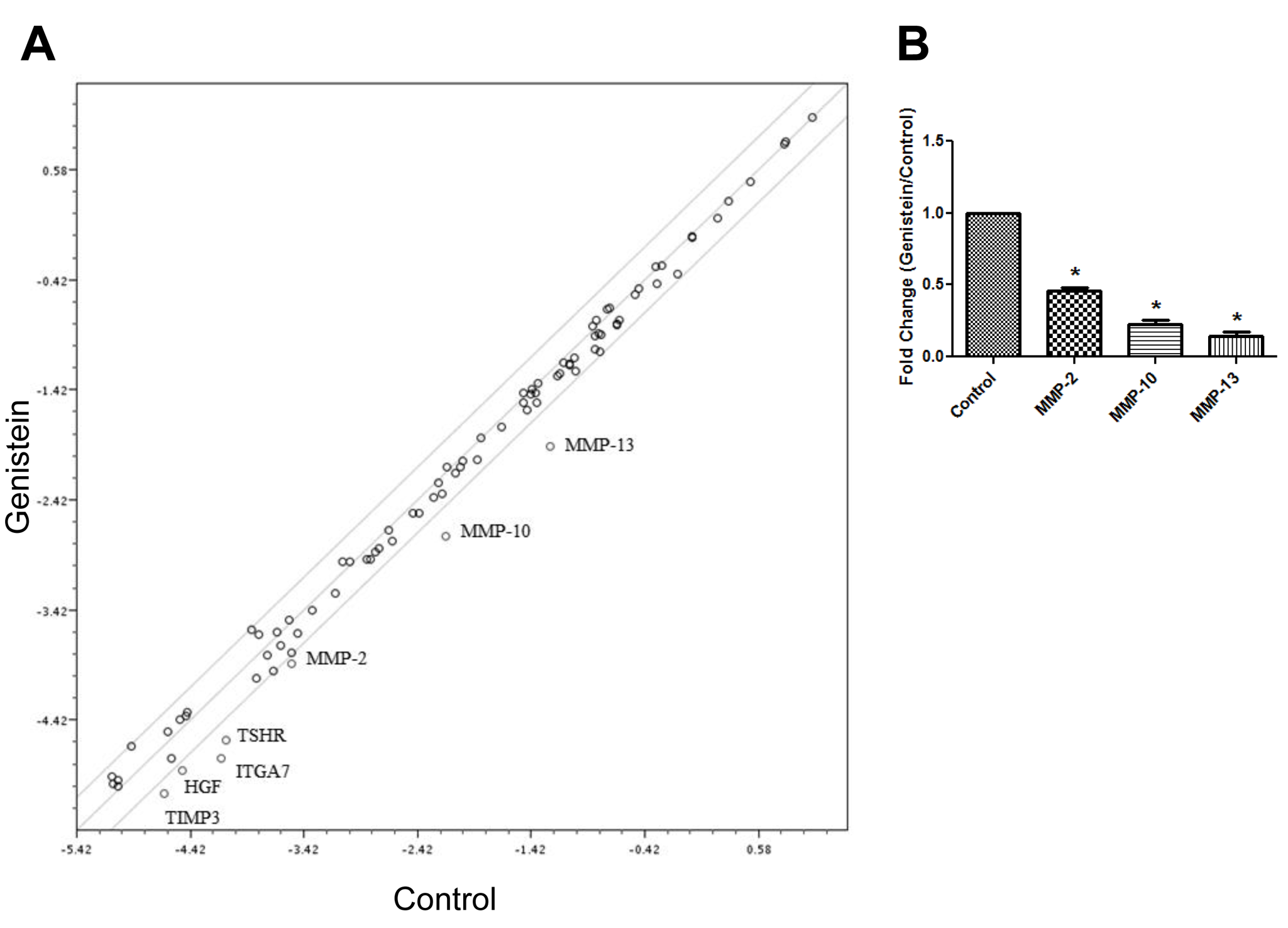

Supplement: Figure S1 — Extracellular Matrix and Adhesion Molecules RT2 Profiler Array. A) PC3-M cells were treated with 10 µM genistein or DMSO control for 3 days, and the Extracellular Matrix and Adhesion Molecules RT2 Profiler PCR Array performed as per manufacturer's instructions three separate times. A ≥2 fold change with a two-sided Student's t-test p value ≤0.05 was considered significant. Seven genes of interest were identified: HGF, ITGA7, MMP-2, MMP-10, MMP-13, TIMP3, TSHR. B) Confirmation of MMP-2, MMP-10, and MMP-13 regulation by genistein by qRT/PCR. Cells were treated with 10 µM genistein or DMSO control for three days, and qRT/PCR performed using gene-specific primer/probe sets. Data are from three experiments, each in replicates of N = 2. * denotes p≤0.05 between control and experimental group. (TIF) [file pone.0102289.s001.tif]

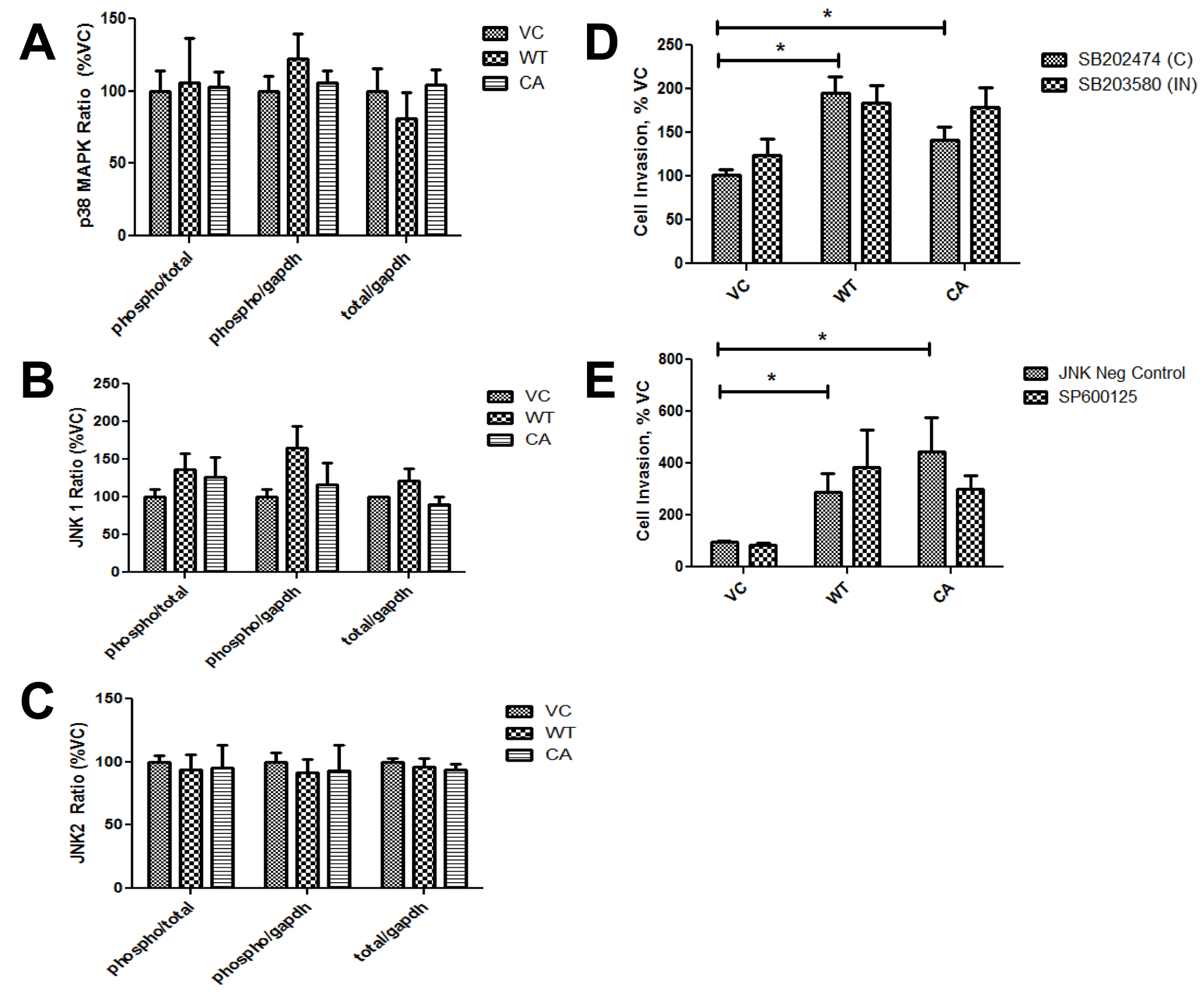

Supplement: Figure S2 — Protein expression of p38 MAPK and JNK, Cellular invasion with p38 MAPK and JNK inhibitors. A-C) The phosphorylated and total protein levels of p38 MAPK, JNK1, and JNK2 as measured by western blot. These were performed in triplicate, quantified, and represented as mean ± SE. D-E) Relative cellular invasion following 48 treatment with chemical inhibitors SB203580 or SP600125, which inhibit p38 MAPK and JNK respectively, or their respective negative controls, SP202474 or JNK Negative Control II (N1-Methyl-1,9-pyrazoloanthrone). Data are from three experiments, each in replicates of N = 3. * denotes p≤0.05 between the indicated groups. (TIF) [file pone.0102289.s002.tif]
